# Rapid Prediction of Multidrug-Resistant Klebsiella pneumoniae through Deep Learning Analysis of SERS Spectra

**DOI:** 10.1128/spectrum.04126-22

**Published:** 2023-03-06

**Authors:** Jing-Wen Lyu, Xue Di Zhang, Jia-Wei Tang, Yun-Hu Zhao, Su-Ling Liu, Yue Zhao, Ni Zhang, Dan Wang, Long Ye, Xiao-Li Chen, Liang Wang, Bing Gu

**Affiliations:** a Department of Laboratory Medicine, School of Medical Technology, Xuzhou Medical University, Xuzhou, Jiangsu Province, China; b Laboratory Medicine, Guangdong Provincial People’s Hospital (Guangdong Academy of Medical Sciences), Southern Medical University, Guangzhou, Guangdong Province, China; c Laboratory Medicine, The Affiliated Xuzhou Infectious Diseases Hospital of Xuzhou Medical University, Xuzhou, Jiangsu Province, China; d Department of Intelligent Medical Engineering, School of Medical Informatics and Engineering, Xuzhou Medical University, Jiangsu Province, Xuzhou, China; e Laboratory Medicine, The Second Affiliated Hospital of Xuzhou Medical University, Xuzhou, Jiangsu Province, China; f School of Medical and Health Sciences, Edith Cowan University, Joondalup, Western Australia, Australia; American Type Culture Collection

**Keywords:** Raman spectroscopy, *Klebsiella pneumoniae*, multidrug resistance, carbapenem, polymyxins, deep learning

## Abstract

Klebsiella pneumoniae is listed by the WHO as a priority pathogen of extreme importance that can cause serious consequences in clinical settings. Due to its increasing multidrug resistance all over the world, K. pneumoniae has the potential to cause extremely difficult-to-treat infections. Therefore, rapid and accurate identification of multidrug-resistant K. pneumoniae in clinical diagnosis is important for its prevention and infection control. However, the limitations of conventional and molecular methods significantly hindered the timely diagnosis of the pathogen. As a label-free, noninvasive, and low-cost method, surface-enhanced Raman scattering (SERS) spectroscopy has been extensively studied for its application potentials in the diagnosis of microbial pathogens. In this study, we isolated and cultured 121 K. pneumoniae strains from clinical samples with different drug resistance profiles, which included polymyxin-resistant K. pneumoniae (PRKP; *n* = 21), carbapenem-resistant K. pneumoniae, (CRKP; *n* = 50), and carbapenem-sensitive K. pneumoniae (CSKP; *n* = 50). For each strain, a total of 64 SERS spectra were generated for the enhancement of data reproducibility, which were then computationally analyzed via the convolutional neural network (CNN). According to the results, the deep learning model CNN plus attention mechanism could achieve a prediction accuracy as high as 99.46%, with robustness score of 5-fold cross-validation at 98.87%. Taken together, our results confirmed the accuracy and robustness of SERS spectroscopy in the prediction of drug resistance of K. pneumoniae strains with the assistance of deep learning algorithms, which successfully discriminated and predicted PRKP, CRKP, and CSKP strains.

**IMPORTANCE** This study focuses on the simultaneous discrimination and prediction of Klebsiella pneumoniae strains with carbapenem-sensitive, carbapenem-resistant, and polymyxin-resistant phenotypes. The implementation of CNN plus an attention mechanism makes the highest prediction accuracy at 99.46%, which confirms the diagnostic potential of the combination of SERS spectroscopy with the deep learning algorithm for antibacterial susceptibility testing in clinical settings.

## INTRODUCTION

Carbapenem-resistant *Enterobacteriaceae* (CRE) are described by WHO as priority pathogens of “extreme importance” and can cause serious and difficult-to-treat infections, with Klebsiella pneumoniae having the highest incidence, accounting for 57 to 74% of all CRE infections (1–3). Currently, the treatment options for carbapenem-resistant K. pneumoniae (CRKP) infections are very limited. Polymyxins (polymyxin B and colistin, differing by one amino acid in the peptide ring) are considered the last-line treatment for multidrug-resistant (MDR) bacterial infections (4), which is generally reserved for salvage therapy of CRKP (5). However, considering the nephrotoxicity and neurotoxicity of polymyxins, blind drug use is not advisable. In a cohort study that was nested within the Consortium on Resistance against Carbapenems in K. pneumoniae (CRACKLE) by Rojas et al., it was found that 13% of CRKP was also colistin resistant, and increased mortality was observed for corresponding patients (6). Therefore, it is extremely important for the laboratory to quickly and accurately diagnose CRKP and polymyxin-resistant K. pneumoniae (PRKP) in parallel (7).

Until now, clinical laboratories do not list Gram-negative bacteria polymyxin resistance (PR) as a routine drug susceptibility testing, which may lead to the underdiagnosis of PR bacteria, e.g., PRKP, and then facilitate their transmission (8). Clinical diagnosis of PR bacteria often requires the duration of 3 to 4 days or more time, and the difficulties in PR testing are mainly caused by the cationic properties and poor diffusions of polymyxins into the agar plate and the presence of heteroresistance in many pathogens (9). The broth dilution method (BDM) is currently the only PR testing method recommended by the Clinical and Laboratory Standards Institute (CLSI), and it is time-consuming and labor intensive (10). The Kirby-Bauer (K-B) procedure and Etest strips are convenient and inexpensive methods and do not require specific equipment, but these methods suffer from high false-negative rates (11). Other rapid screening methods, e.g., rapid polymyxin NP test (2 h), are required to detect bacterial growth in the presence of a known polymyxin concentration (12), while PCRs cannot fully reflect the drug-resistant phenotypes (13, 14).

The Raman scattering (RS) effect is an inelastic scattering of photons that could interact with molecules in material samples, which could then form a unique fingerprint to represent the material by using Raman spectrometer (15). However, regular RS spectroscopy suffers from a low signal-to-noise ratio (SNR), which makes the method hardly applicable in real-world settings. In 1970s, an advanced RS technique known as surface-enhanced Raman scattering (SERS) was developed with high capture signals that broke fresh ground for the application of RS (16). So far, various studies have confirmed that SERS, as a label-free, noninvasive, and low-cost technique with high SNR, holds the potential to rapidly discriminate bacteria at different division levels and recognize bacteria with different antibiotic-resistant profiles (17, 18). During the performance of SERS, metallic nanoparticles were needed as the substrates for the enhancement of SNR, and silver, copper, and gold are the three most commonly used metals for SERS-based methods, which have achieved limits of detection (LODs) in the order of 10 CFU/mL (17). In particular, label-free SERS for the detections of bacterial species and antibiotic resistances does not require any specific antibodies (19), which, therefore, is potentially suitable for routine clinical diagnosis and screening (20, 21).

It should be noted that although SERS technique provides unique molecular fingerprints for samples such as bacteria and antibiotics, etc., spectral differences were really subtle, especially for bacteria at the subspecies level with different antibiotic resistance profiles (22). In addition, SERS spectral data have the characteristics of high dimension and poor reproducibility (23), which requires a large number of spectra for each sample to confirm spectral signatures, complicating data classification and sample prediction. Therefore, regular statistical analysis cannot meet SERS spectral processing procedures, while various machine learning techniques have been adopted to automatically and efficiently analyze these data (15, 24, 25). In this study, we isolated 50 strains of carbapenem-sensitive K. pneumoniae (CSKP), 50 strains of CRKP, and 21 strains of PRKP from clinical samples. All SERS spectra were processed to calculate the average Raman spectrum of each strain and the corresponding characteristic peaks, while the difference between the common characteristic peaks of each average spectral curve was calculated using statistical analysis methods, and the characteristic peaks with statistical differences were selected. To effectively distinguish three different K. pneumoniae species, we adopted the deep learning algorithm convolutional neural network (CNN) and a CNN-attention model that uses an attention mechanism in tandem with CNN. Performance of the two models was compared in terms of accuracy, sensitivity, and specificity, and it was concluded that the CNN-attention model had better performance in distinguishing the three K. pneumoniae strains. In addition, we used the Grad-CAM algorithm to visualize the significance heatmap of the CNN-attention model on each strain sample, which effectively explained how the deep learning model calculated and identified the SERS spectral curve. Taken together, this study successfully discriminated SERS spectra of clinical CSKP, CRKP, and PRKP strains with deep learning algorithms, which confirms that the deep learning algorithm combined with SERS spectroscopy holds significant potentials in antibiotic resistance diagnosis for bacterial pathogens in the future.

## RESULTS AND DISCUSSION

The SERS data set was constructed based on three K. pneumoniae groups, that is, CSKP (*n* = 50), CRKP (*n* = 50), and PRKP (*n* = 21), in which each K. pneumoniae strain corresponded to 64 reproducible SERS spectra. Therefore, a total of 7,744 SERS spectra were collected for downstream analysis. All the K. pneumoniae strains included in this study were confirmed through clinical diagnosis, biochemical tests, and matrix-assisted laser desorption ionization–time of flight mass spectrometry (MALDI-TOF MS) validations. The results of the drug susceptibility profiles for all the strains are presented in Fig. S1 in the supplemental material. In order to get a direct understanding of K. pneumoniae strains, colony morphologies were recorded on Columbia blood agar plates (Fig. S2A to C), which were then tested for antibiotic resistance to ertapenem (Fig. S2D to F) and polymyxin B (Fig. S2G to I) by Liofilchem MIC test strips (MTS). Complete SERS spectra of K. pneumoniae strains used in this study are publicly available at the SERS Spectral Database of MRKP strains (MRKP-SSD) website, http://139.9.193.178/mrkp-ssd/#/home, which is freely accessible with a relevant introduction to MRKP. As it is an open-access platform, users can download all the SERS spectral data and can also upload their own SERS spectra for drug resistance prediction in terms of carbapenem and polymyxin resistance in K. pneumoniae.

### AgNPs showed better SERS effects than AuNPs for K. pneumoniae strains.

**FIG 1:**
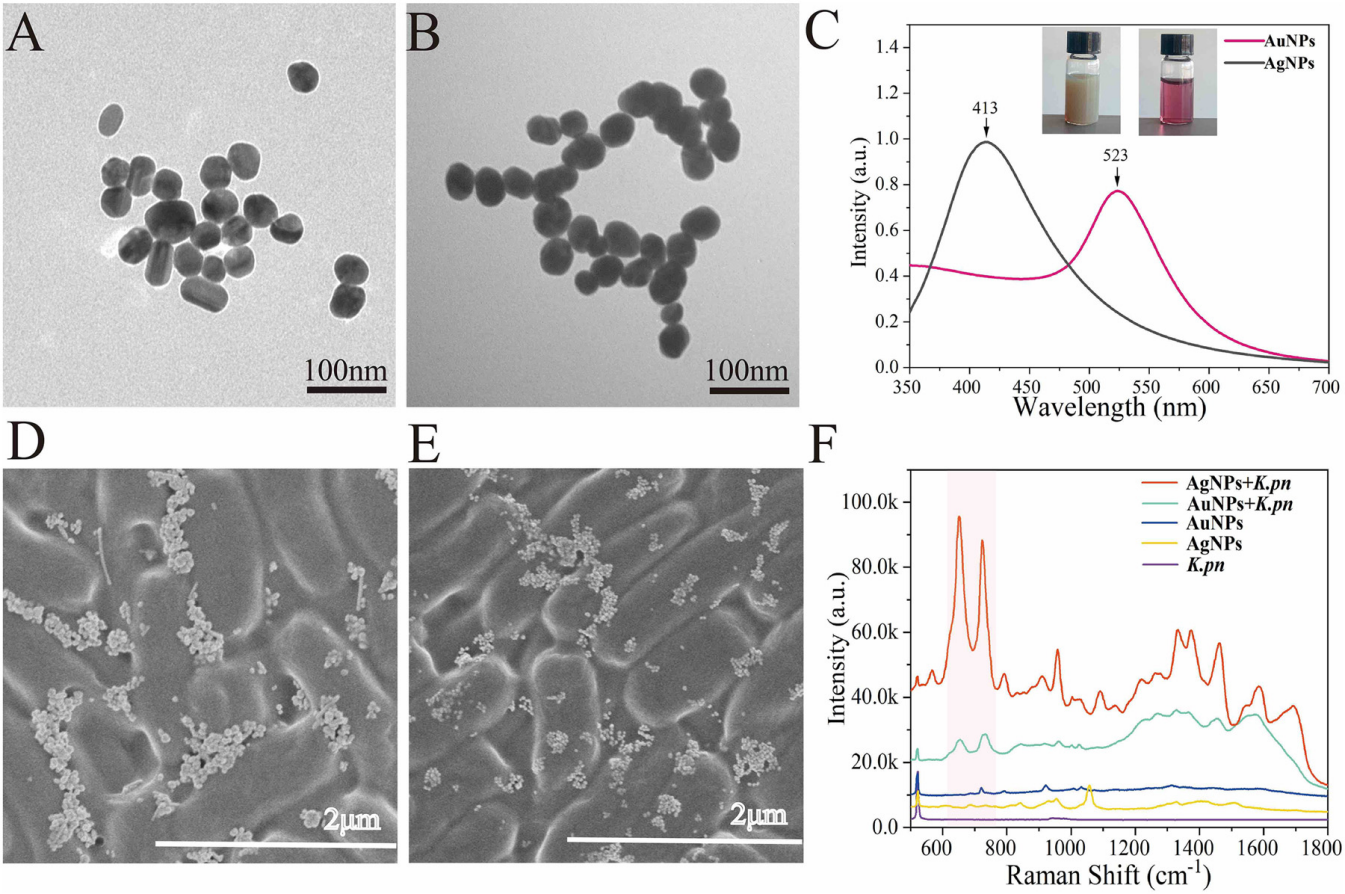
Metallic nanoparticle structural characterization and their signal enhancement capacities in Raman scattering effect. (A) TEM of AgNPs; (B) TEM of AuNPs; (C) UV-Vis of AgNPs and AuNPs; (D) SEM of AgNPs plus K. pneumoniae; (E) SEM of AuNPs plus K. pneumoniae; (F) Raman spectra of pure liquid culture, AgNPs, AuNPs, K. pneumoniae (*K.pn*) plus AgNPs, and K. pneumoniae plus AuNPs.

In this study, we compared the effects of silver nanoparticles (AgNPs) and gold nanoparticles (AuNPs) in terms of their effects in SERS spectroscopy when mixed with K. pneumoniae strains (AgNPs, 10 mmol/L; AuNPs, 10 mmol/L). The structures of the two nanoparticles were characterized via transmission electron microscopy (TEM) and UV-visible (UV-Vis) spectroscopy. TEM images of the AgNPs and AuNPs are shown in Fig. 1A and B. The results indicated that the as-prepared AgNPs and AuNPs were monodispersal, and the average diameters were in the range of 40 to 45 nm. The UV-Vis absorption spectrum showed that the absorption peak of AgNPs (gray line) was located at 413 nm, while the peak of AuNPs (wine-red line) was at 523 nm (Fig. 1C), which was consistent with previous reports by Balavigneswaran and Hernández et al. (26, 27). The zeta potentials of AgNPs and AuNPs were −25.7 mV and −23.1 mV, respectively. In addition, we also conducted scanning electron microscopy (SEM) analysis of the mixtures of K. pneumoniae-AgNPs and K. pneumoniae-AuNPs after natural drying on a silicon wafer, respectively, through which rod-shaped K. pneumoniae could be seen with scattered and agglomerated particles surrounding, providing a SERS hot spot for signal enhancement (Fig. 1D and E). Finally, SERS spectra were generated for pure liquid culture, AgNPs, AuNPs, K. pneumoniae plus AgNPs, and K. pneumoniae plus AuNPs, the results of which are demonstrated in Fig. 1F. According to the results, K. pneumoniae plus AgNPs provided stronger SERS signal than K. pneumoniae plus AuNPs. Therefore, for the following studies, only AgNP was used as the substrate for all the SERS analysis.

### SERS spectroscopy showed concentration-dependent effects.

**FIG 2:**
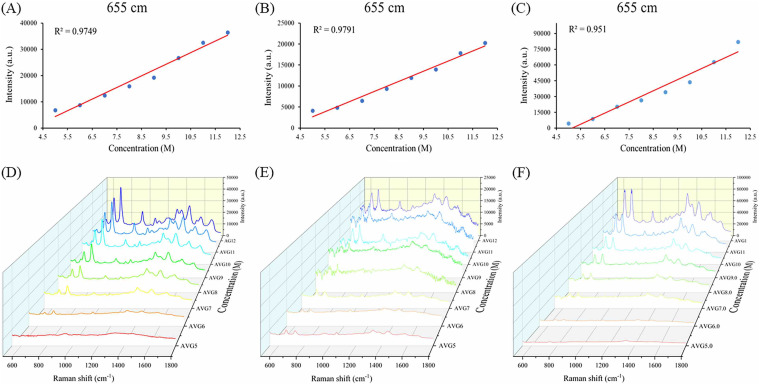
Illustration of SERS spectra in a linearly dose-dependent manner. (A to C) Linear relationship between bacterial concentration and SERS signal intensity for CSKP, CRKP, and PRKP, respectively. (D to F) SERS spectra for CSKP, CRKP, and PRKP at the concentrations ranging from 5 M to 12 M (M, McFarland standard).

The McFarland turbidimeter is a common instrument in clinical laboratory for measuring bacterial concentrations in liquid culture (28). When bacterial concentrations of CSKP, CRKP, and PRKP were above a 5.0 McFarland standard, the concentrations had a linear relationship with SERS signal intensities (Fig. 2A to C), which suggested good reproducibility of SERS spectroscopy, while the number of characteristic peaks was also gradually increased with the increment of SERS intensities (Fig. 2D to F). For the label-free bacterial spectrum, the appearance of some characteristic peaks does not prove to be K. pneumoniae because of the common lipids, carbohydrates, proteins, and other components on the bacterial surface (29, 30). Therefore, a complete fingerprint spectrum is important for bacterial identification. We showed the changes in the peaks and intensities of the SERS spectra of K. pneumoniae strains at different concentrations and found that at concentrations of 5.0 McFarland standard and above, the number of characteristic peaks was stable, and peaks with lower intensities were also present, which was necessary for the establishment of fingerprint profiles of our entire SERS data set. The development of machine learning systems with complex, high-dimensional, and sparse data in previous studies had paved the way for SERS single-cell detection and drug resistance analysis. However, SERS fingerprints obtained from low concentrations of bacterial copy numbers were still not practiced yet in routine laboratories (31).

### Average Raman spectra and characteristic peaks.

**FIG 3:**
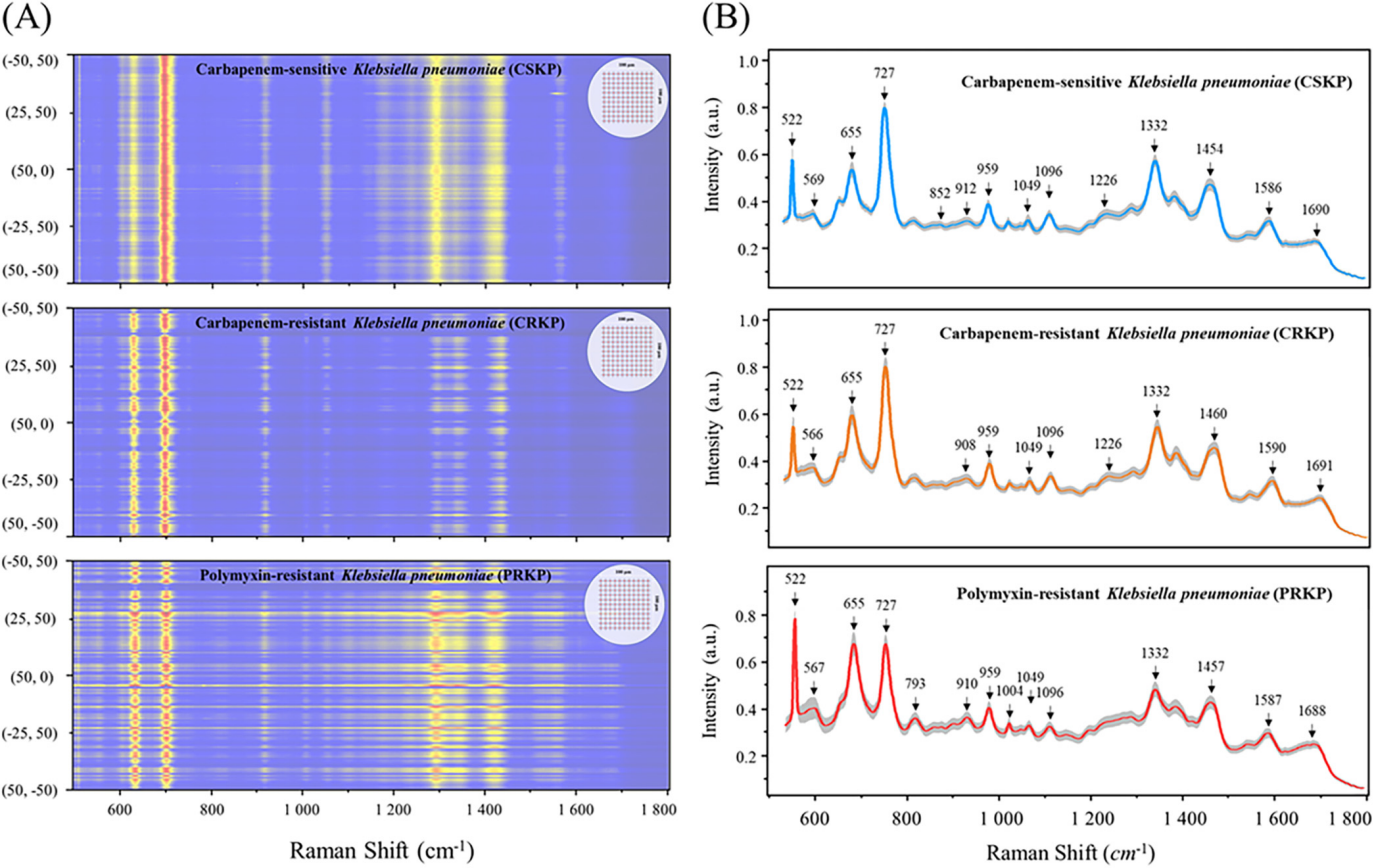
Schematic illustration of SERS spectra across surface areas and averaged SERS spectra of CSKP, CRKP, and PRKP. (A) Heatmap plot of SERS spectra for representative CSKP, CRKP, and PRKP strains showing enhancement consistency across a surface area of 100 by 100 μm^2^. The inset on the upper right corner shows the *xy* map region scanned on the silicon wafer. (B) Average SERS spectra and the corresponding characteristic peaks of CSKP (*n* = 3,200), CRKP (*n* = 3,200), and PRKP (*n* = 1,344) isolated from clinical samples.

In order to investigate the regional SERS signals when AgNPs mixed with bacterial samples (concentration, 5 M), we collected 121 SERS spectra across a 100- by 100-μm^2^ region (step = 10 μm, *x* = 11, *y* = 11) within a dried sample spot on the silicon wafer for each of representative CSKP, CRKP, and PRKP strains by using the map image acquisition function with rectangle-filled mode of the Raman spectrometer (Fig. 3A). The results showed that SERS spectra with good consistency were obtained in the area, though at some points, there were no signals, which should be caused by the absence of bacteria during scanning. The SERS signals obtained at all the points showed corresponding characteristic peak shifts. Considering the coffee ring effect, the signal intensity and quality of the acquired signals in the entire area were acceptable. In order to better understand SERS spectral differences of the three groups of K. pneumoniae strains, we generated averaged SERS spectra for CSKP (*n* = 3,200), CRKP (*n* = 3,200), and PRKP (*n* = 1,344) by calculating the average signal intensity for each Raman shift, respectively, while a standard 20% standard error band (SEB) was added to each averaged SERS spectrum to reflect SERS spectral reproducibility (Fig. 3B).

**FIG 4:**
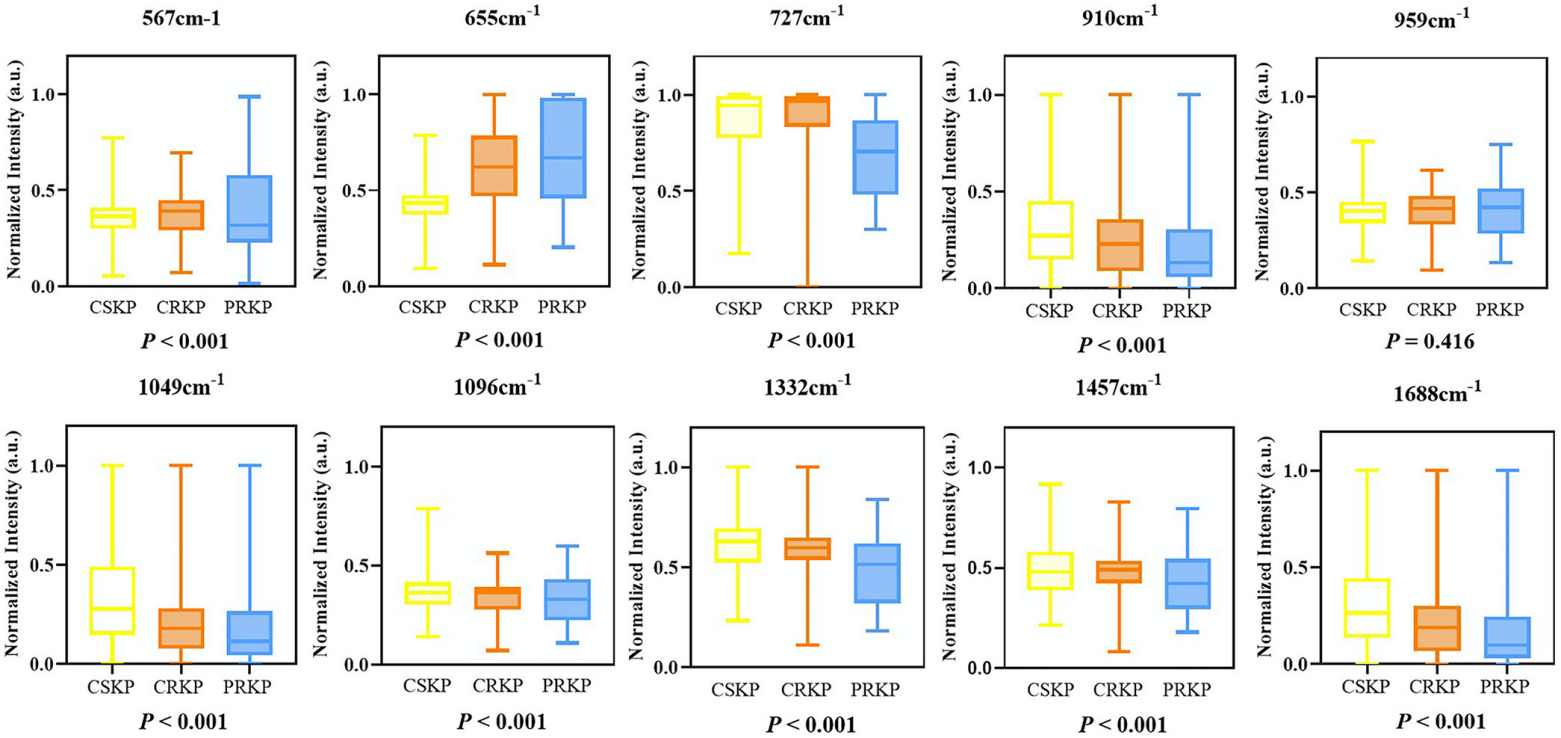
Boxplots for normalized intensities of SERS spectra for CRKP, CSKP, and PRKP at representative Raman shifts corresponding to important biological components. A *P* value of <0.05 is considered statistically significant.

Further analysis of the average SERS spectra via the software LabSpec revealed that each K. pneumoniae group had its own specific combination of characteristic peaks that are marked with black arrows in Fig. 3B, which corresponded to specific metabolites and are summarized in Table 1. By comparing the characteristic peaks in the average Raman spectra among the three K. pneumoniae groups, it was revealed that characteristic peaks were either present in all the groups or biasedly present in certain groups. For example, all the SERS spectra had a strong Raman shift peak at 522 cm^−1^, which corresponded to the silicon substrate due to the use of silicon wafer (32). Other shared SERS bands represented carbohydrates, saccharides, proteins, lipids, and DNA components common to the three groups of K. pneumoniae (33–39). Peaks at 852 cm^−1^ and 1,586 cm^−1^ (40, 41) were uniquely present in the average SERS spectrum of CSKP, which indicated that tyrosine and phenylalanine may change when carbapenem resistance developed. Peaks at 1,589 to 1,590 cm^−1^ showed that the ring mode of adenine and guanine stretched in CRKP and PRKP (20, 33). As for PRKP, unique peaks at 793 cm^−1^ and 1,004 cm^−1^ were identified but were absent at 1,226 cm^−1^ (42–44). In addition, we also applied statistical methods to analyze characteristic peaks of biological components shared by CSKP, CRKP, and PRKP to estimate the compositional differences of K. pneumoniae among the three groups (Fig. 4). Here, the lower limit and upper limit of the boxes represented the first and third quartiles, and the middle line represents the second quartile (median). The results showed that the overall distribution and median of all characteristic peaks except at 959 cm^−1^ were significantly different according to the *P* value (less than 0.05 means the difference is statistically significant). PRKP strains we collected all showed polymyxin resistance on the basis of CRKP, which can be regarded as the deepening of the resistance range of CRKP. At the Raman shift of 655 cm^−1^, the median of normalized intensities showed an upward trend, which indicates that among CSKP, CRKP, and PRKP, the C-C and C-S bonds of the proteins were stretching more and the deformation of tyrosine was intensified, and the content of amino acid and guanine was also increasing. Proteins and lipids of K. pneumoniae may be decreased at 1,688 cm^−1^. Proteins, DNA, NADH, and NH_2_ stretching (adenine, polyadenine, and DNA) and amide I were also increased. In general, adenine, flavin formation of glycosidic ring patterns, adenine ring respiration patterns, and phosphodiester extensions in DNA and out-of-plane ring respiration patterns of tyrosine and lipids may be the factors causing carbapenem resistance and polymyxin resistance in K. pneumoniae. Therefore, the main reason for the changes of polymyxin resistance could be due to the modification of cell membrane lipopolysaccharide, which might be an important marker to distinguish CSKP, CRKP, and PRKP in clinical diagnosis (45). Taken together, Raman spectroscopy has a good predictive effect on the changes of bacterial resistance components.

**TABLE 1:** Characteristic peaks identified in the average SERS spectra of CSKP, CRKP, and PRKP strains and their corresponding chemical components^*a*^

RS (cm^−1^)	Band assignment(s)	CSKP	CRKP	PRKP	Reference
566, 567, 569	β(C-C-C), β(C-C-O), β(O-C-O) (540–595)				51
655	C-S str, C-C tw of proteins tyrosine def, (COO^−^) in amino acids, guanine (645–660)				40
727	C-S/cysteine (726), adenine, glycosidic ring mode from flavin (FAD, NAD, ATP, DNA) (724)				40, 52
793	NH_2_ wagging				43
852	‘‘Buried’’ tyrosine				42
908, 910, 912	Saccharides/proteins (906–911)				53
959	(C-N) str (956), polysaccharide				54, 55
1004	Tyrosine/tryptophane (symmetric ring C-C str), phenylalanine				42, 44
1049	Proteins/lipids (1050), C-O str (1046)				53, 54
1096	DNA, C-O str (1098)				55
1226	Proline (C-N str), amide III random, lipids				33, 44
1332	C-H def, proteins/DNA/NADH, NH_2_ str (adenine, polyadenine, and DNA)				51, 53
1457	C-H_2_ def (1440–1460), δ(CH_2_) saturated lipids, CH_2_ def/CH def/CH_2_ scissors (1455)				20, 40, 42
1586	Phenylalanine, amide II (1585), C=C (1587)				40, 41
1589, 1590	Adenine, guanine (ring str), CC str + NH_2_ bend (1590)				33, 39
1688, 1690, 1691	Amide I				33

a RS, Raman shift; str, stretching; tw, twisting; def, deformation; FAD, flavin adenine dinucleotide; β, in-plane bending. Grey shading region means the presence of the characteristic peaks in the SERS spectra while blank region means the absence of the characteristic peaks.

### Machine learning analysis of SERS spectra.

The purpose of supervised machine learning analysis is to construct suitable predictive models to identify differences in Raman spectra between different strains. For traditional supervised algorithms, as in the work of Ciloglu et al. (46), when analyzing the difference between the SERS spectra of colistin-resistant and -susceptible strains of K. pneumoniae, two methods of principal-component analysis and autoencoder were used to extract spectral features, which were then combined with support vector machine (SVM) to classify K. pneumoniae; the performance of the autoencoder-SVM algorithm reached an accuracy of 94% (47). As for the deep learning algorithm, Lu and his collaborators applied the CNN algorithm to identify antibiotic resistance genes (ARGs) from K. pneumoniae strains, according to which 94.24 ± 1.14% accuracy was achieved (48). In this study, two deep learning algorithms, CNN and CNN-attention, were recruited to analyze the SERS spectral data of three K. pneumoniae groups. The performance of the two models on the test data set was evaluated by six evaluation metrics, that is, accuracy (ACC), precision (Pre), Recall, F1, 5-fold cross-validation (5-fold CV), and area under the curve (AUC). The results are shown in Table 2. According to the results, the classification accuracy of the CNN model reached 98.23%, while the 5-fold CV score also achieved 96.89%, confirming that the CNN model can better distinguish three different Klebsiella pneumoniae groups. When the attention mechanism was incorporated into the CNN model, the prediction accuracy increased to 99.46%, indicating that the attention mechanism could further improve the ability of the model to extract features, and the 5-fold CV score increased to 99.90%, suggesting that the model became more robust than the CNN model. Therefore, it was concluded that the CNN-attention model could effectively discriminate three groups of K. pneumoniae strains with different resistance profiles, which surpassed all previous methods.

**TABLE 2:** Comparative analysis of the predictive capabilities of the two deep learning algorithms, CNN and CNN-attention, on SERS spectra data belonging to CSKP, CRKP, and PRKP

Algorithm	ACC (%)	Precision (%)	Recall (%)	F1 (%)	5-Fold CV (%)	AUC (%)
CNN	98.23	97.76	97.87	97.62	96.89	97.70
CNN-attention	99.46	97.91	97.54	97.64	98.87	99.11

**FIG 5:**
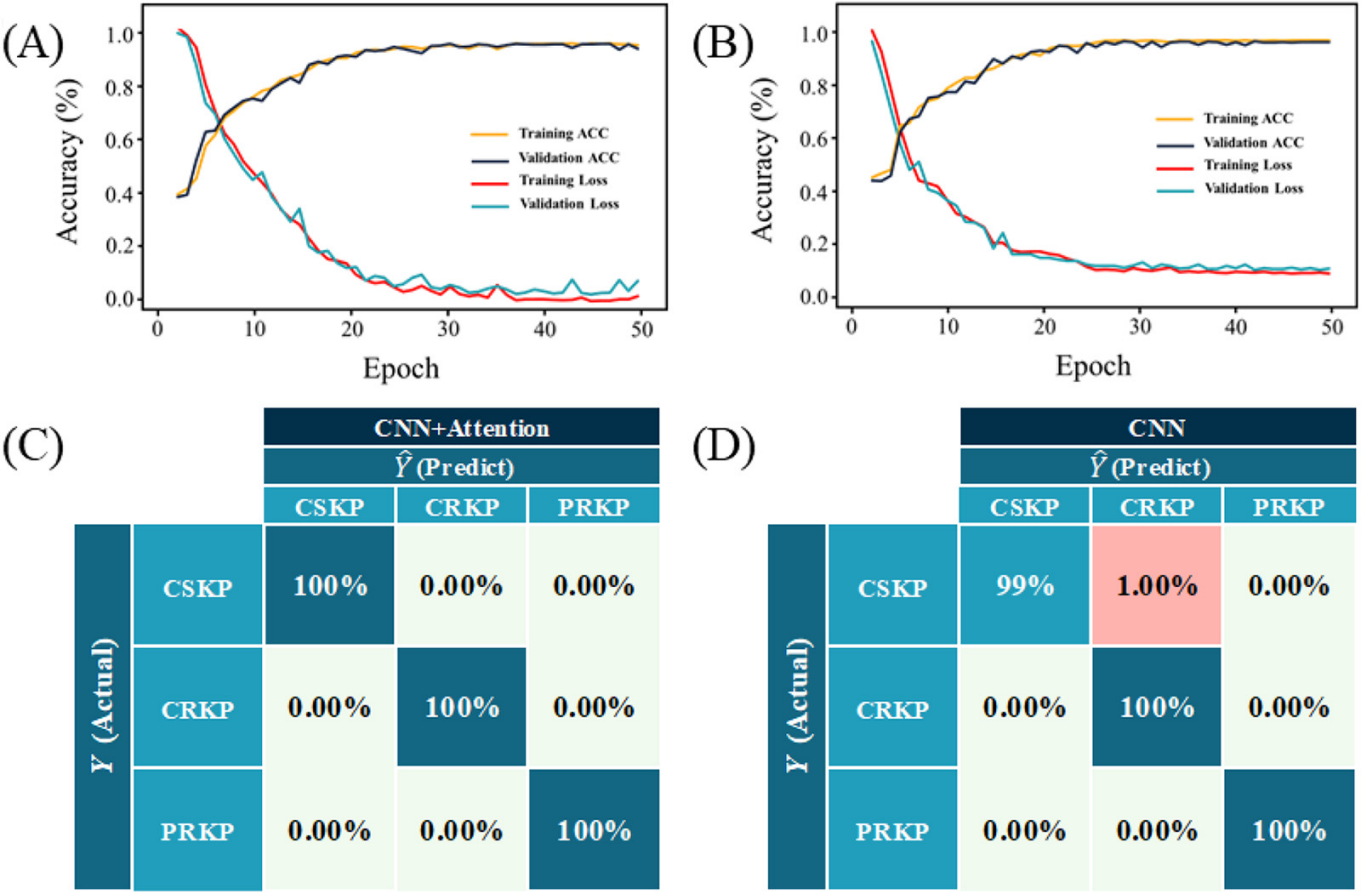
Performance evaluations and confusion matrices of the two deep learning algorithms CNN and CNN-attention. (A) Learning curve and loss curve for CNN-attention model. (B) Learning curve and loss curve for CNN model. (C) Confusion matrix for CNN-attention model. (D) Confusion matrix for CNN model.

In order to gain a better understanding of the model performance, we generated the learning curve and loss curve of the CNN and CNN-attention models, respectively (Fig. 5A and B). It could be seen that since the learning accuracy was close to 100% on both the training set and the validation set for both models, and as the number of model iterations (epochs) increased to 30, the loss value reached the plateau period. In order to know the specific predictions of the CNN and CNN-attention models for different groups of K. pneumoniae, the confusion matrix was used as a quantitative visualization method, which aimed to describe the relationship between the real distribution of sample data and the predicted results in matrix form. The results in Fig. 5C and D show that the CNN-attention model could completely identify the test data set correctly, with an average recognition accuracy of 100%, while the CNN model had 100% accuracy in identifying CRKP and PRKP strains. When identifying the CSKP strain, 1% of CSKP spectral data were misidentified as CRKP, which may be due to the high similarities between the two groups of K. pneumoniae.

### Interpretability of deep learning analysis.

**FIG 6:**
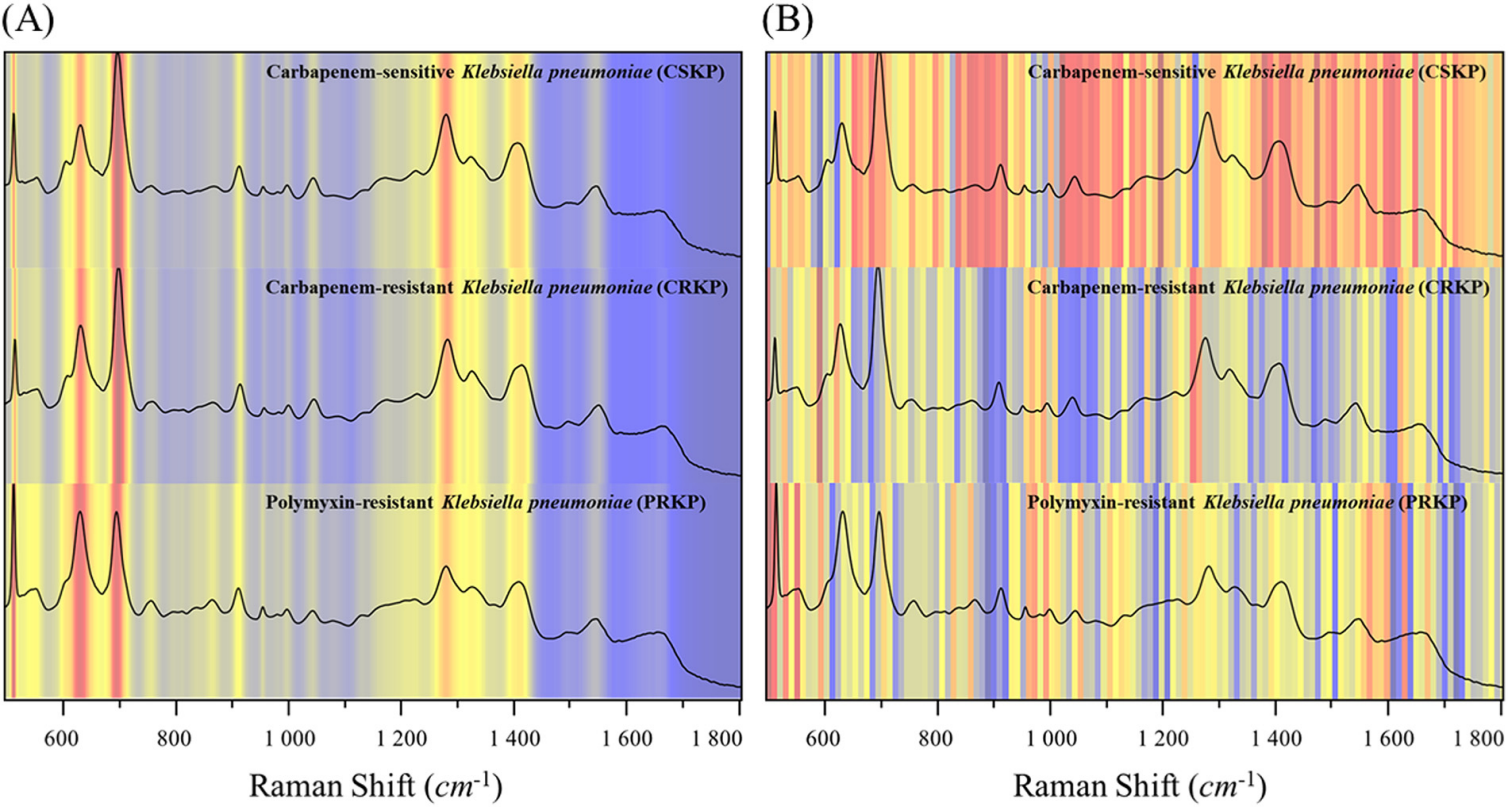
Heatmap of SERS spectra before and after inputting into the deep learning model. (A) Raw SERS spectra. (B) Model-processed SERS spectra. The spectral regions that played key roles in different K. pneumoniae groups were partially different with overlap regions, which also explained why the deep learning models could partially misidentify the SERS spectra.

In the field of data analysis, decision-making systems should provide clear evidence to explain the results. However, the black box nature of deep learning makes model interpretability difficult. Therefore, understanding how the deep learning network designed in the study classifies the spectrum is crucial for evaluating the network performance and interpretability of the study model. The Grad-CAM algorithm proposed by Selvaraju in 2017 enables the “interpretability” of many CNN-based classification models (34). Once the algorithm was proposed, it was quickly applied in ore identification, cell microbial identification, and clinical pathogen detection (35–37). For example, Deng et al. used the Grad-CAM algorithm to observe the difference between the models in distinguishing different spectra of bacterial isolates, providing a reference for further analysis of bacterial spectra at the biomolecular level (38). Therefore, in order to observe the distribution of model classification weights in the spectrum, the parts with higher classification weights in the full spectrum of the spectrum were analyzed in this study. In addition, Grad-CAM was introduced to reveal the importance of different Raman shifts in the deep learning model during the analysis of three groups of K. pneumoniae strains. We used average Raman spectra as representative spectra for CSKP, CRKP, and PRKP. The raw spectral data were used for the analysis as shown in Fig. 6A, which presented that the heatmap of the raw data was distributed according to the intensity of the characteristic peaks. Spectral regions with high intensities appeared in warm colors (yellow), while those spectral regions with lower intensity appeared in cool colors (blue).

**FIG 7:**
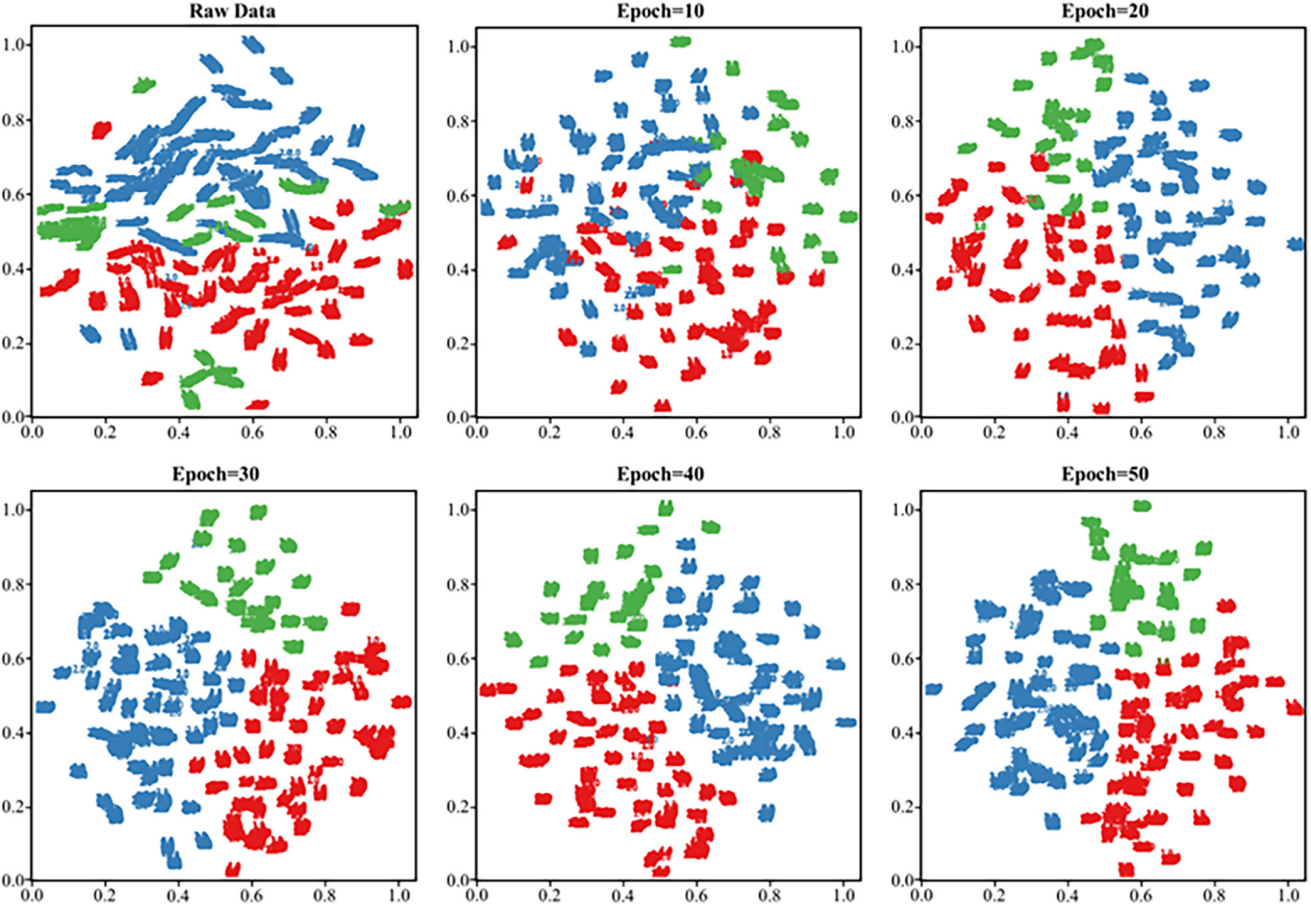
Schematic illustration of how the number of iteration (epoch) influenced the classification effects of deep learning models. As the number of convolutional layers and the number of iterations continued to increase, the data of different categories changed from the original mixed state to the gradual separation. After epoch reached to 30, the recognition of the three K. pneumoniae groups by the model gradually became stable.

For the spectral data after inputting into the model, the Grad-CAM results showed that different groups of K. pneumoniae models focused on different spectral feature intervals, and they were not completely classified according to the characteristic peaks as we inferred in Fig. 6B. For example, within the Raman displacement range of 950 to 1,300 cm^−1^, the model paid more attention to CRKP but did not assign more weight to CSKP. In the 1,250- to 1,280-cm^−1^ region, the attentional heatmap for CRKP was shown in dark orange, while in CSKP, it was completely dark blue. In the PRKP group, it was found that in the range of 860 to 930 cm^−1^, the model did not assign much weight to most spectral features, while in CSKP, it attracted high attention. The same was also shown in the Raman shift interval between 1,680 and 1,740 cm^−1^. These significant differences suggested that our model could detect subtle differences in the spectra among different K. pneumoniae groups. Therefore, the identification model could be used as a reference when distinguishing the SERS spectra of K. pneumoniae with different profiles of antibiotic resistance. To demonstrate the process of how deep learning models classified the three groups of K. pneumoniae strains, we used t-distributed stochastic neighbor embedding (t-SNE) to depict the classification results of the SERS spectra. It could be seen that as the epochs increased, the model classification effect was gradually improved until the epoch reached 30, which was also consistent with the learning curve of our model in Fig. 7. When epoch was equal to 30, it could be seen that the SERS spectral data for each group of K. pneumoniae were clustered together, shown as numbers labeled with different colors, and the different groups were relatively separated from each other. In sum, we mixed colloidal silver nanoparticles with the bacterial solutions to simply acquire SERS spectra from 121 clinical K. pneumoniae strains, which were then combined with the deep learning algorithm CNN-attention mechanism to distinguish CSKP, CRKP, and PRKP strains at the accuracy of 100%.

### Conclusion.

In this study, by combining SERS spectroscopy and the deep learning algorithm CNN-attention mechanism, we achieved rapid and accurate discrimination of three groups of clinically important K. pneumoniae strains based on the subtle differences of SERS spectra that were attributed to variations of nucleic acids, proteins, and lipopolysaccharides. Therefore, the SERS technique coupled with deep learning analysis opens up a new direction toward noninvasive, low-cost, operational, and fast-paced diagnosis of K. pneumoniae strains, which holds the potential in large-scale routine use in clinical settings, especially for remote areas with insufficient medical resources. Taken together, computation-assisted label-free SERS spectroscopy showed high specificity and sensitivity during prediction and classification of antibiotic-resistant phenotypes of K. pneumoniae, which lay the foundation for its applications in the rapid profiling of bacterial antibiotic resistance in future.

## MATERIALS AND METHODS

### Collection of K. pneumoniae strains.

CSKP (*n* = 50), CRKP (*n* = 50), and PRKP (*n* = 21) were isolated from clinical samples at the Clinical Microbiology Laboratory of Guangdong Provincial People’s Hospital (Guangzhou Academy of Medical Sciences) and the second Affiliated Hospital of Xuzhou Medical University in China from January 2021 to December 2021. All the strains were cultured on Columbia blood agar plates (Guangzhou Detgerm Microbiological Science, China) at 35°C in the CO_2_ incubator for 24 ± 2 h (Thermo Fisher, USA). All bacteria were confirmed by MALDI-TOF MS (Vitek MS, bioMérieux, France) as K. pneumoniae. A well-growing single colony was first selected and applied to the MALDI-TOF MS target plate. Once the sample was dried, 1 μL of CHCA (α-cyanogen-4-hydroxycinnamic acid) was added onto the sample to dry naturally at room temperature. Escherichia coli ATCC 8739 was used as the quality control strain. Spectra were obtained in the RUO mode with a laser frequency of 75 Hz, 100 shots, and a 2,000- to 20,000-Da mass-charge ratio and analyzed through SARAMIS software. Finally, all the confirmed K. pneumoniae isolates were stored at −80°C in 25% glycerol stocks (Beijing Solarbio Science & Technology Co., Ltd., China) for long-term use.

### Profiling of K. pneumoniae antibiotic resistance.

Liofilchem MIC test strips (Etest) of ertapenem, imipenem, and polymyxin B were used for preliminary screening of CRKP and PRKP. The MICs for 21 antimicrobial agents (ceftazidime-avibactam, tigecycline, polymyxin B, ampicillin, ampicillin/sulbactam, piperacillin-tazobactam, cefazolin, cefotetan, ceftazidime, ceftriaxone, cefoperazone, aztreonam, ertapenem, imipenem, amikacin, gentamicin, tobramycin, ciprofloxacin, levofloxacin, cefoxitin, and compound sulfamethoxazole) were determined for all isolates using the gold-standard broth microdilution method (BMD) method according to the CLSI M7-A10 standard (49). All experiments were repeated in triplicate in separate experiments. The drug susceptibility results of all strains were interpreted according to CLSI M100-S30 and are shown in Fig. S1 in the supplemental material (10), and the specific MIC values and reference ranges of polymyxin B are given in Table S1. Quality control strains used in this study were Escherichia coli ATCC 25922, Pseudomonas aeruginosa ATCC 27853, and Klebsiella pneumoniae ATCC 700603.

### Preparation and characterization of silver and gold nanoparticles.

The preparations of silver nanoparticles (AgNPs) and gold nanoparticles (AuNPs) were based on a classical and facile chemical reduction method (33, 50). The specific methods and nanoparticle characterization are shown in the supplemental material.

### Determination of K. pneumoniae concentration.

Representative strains of CSKP (no. Y039), CRKP (no. Y28), and PRKP (no. P15) were randomly selected from the sample collection and cultured overnight on Columbia blood agar plates. The differences in colony growth and antimicrobial susceptibility results are shown in Fig. S1 and S2. Double-distilled water (ddH_2_O) and McFarland densitometer DEN-1B (Grant-Bio, UK) were used to adjust the concentration of bacterial solutions from a 0.5 (1.5 × 10^8^ cells/mL) to 12.0 (3.6 × 10^9^ cells/mL) McFarland standard with an ascending interval of 0.5 McFarland standard. 5μl AgNPs and 5 μl *K. pneumoniae* solution were vigorously mixed on a vortex mixer for 5 s, and then pipetted 2.5 μL mixture of AgNPs and bacteria solution with the ratio of 1:1 on the silicon wafer to air dry. For each K. pneumoniae strain at each concentration, 20 positions were randomly selected and scanned in the dried spot for SERS signal acquisition.

### Surface-enhanced Raman spectroscopy.

Five microliters AgNPs and 5 μL K. pneumoniae solution (a single colony mixed with 100 μL ddH_2_O) were vigorously mixed on a vortex mixer for 5 s, 2.5 μL of which was then dropped onto a silicon wafer to dry naturally, and then 64 spots were randomly selected for automatic acquisition of SERS spectra. In particular, map image acquisition (matrix mode) of a Renishau Raman instrument was used to scan points (step = 10 μm, *x* = 8, *y* = 8). A total of 64 points were auto-scanned, and a large range was randomly selected for matrix scanning for each sample. The Raman spectrometer and all the parameters were the same as detailed in “Preparation and characterization of silver and gold nanoparticles.”

### SERS spectral data analysis.

**(i) Average SERS spectra.** By calculating the average intensities under each Raman shift for all the SERS spectra that were generated from the same K. pneumoniae group, that is, CSKP, CRKP and PRKP, the average Raman spectrum was then obtained for each K. pneumoniae group. We used WiRE 5.3 software (Renishaw plc., Gloucestershire, UK) to remove cosmic ray for spectra pretreatment, and then the spectra were normalized by the maximum-minimum normalization method without removing noise and smoothing. The 20% standard deviation (SD) for each K. pneumoniae SERS spectrum was also calculated and visualized as a shaded region around the average SERS spectrum. Average Raman spectra with standard error bands (SEB) were plotted using Origin software (OriginLab, USA). The width of the error bands showed the reproducibility of the Raman spectra of each K. pneumoniae group. The wider the error band, the worse the reproducibility. For details of the procedures, please refer to previous publications by Tang et al. (15).

**(ii) Analysis of characteristic peaks.** In order to distinguish the different bacterial species via their SERS spectra, computational identification of characteristic peaks was conducted on the three average SERS spectra via the software LabSpec6 (Horiba Scientific, Japan), respectively. In specificity, the GaussLoren function was used for the analysis with main parameters set to level, 13%; size, 20; and iteration, 5, while other parameters were set to default. All the fitted characteristic peaks were marked with black arrows and labeled with corresponding Raman shifts in the graph. Biological meanings of all the characteristic peaks were sourced from previous reports in the literature and are summarized in Table 1. The significance of differences between medians was determined using nonparametric Kruskal-Wallis test to evaluate significant differences in spectral data after normalization of characteristic peaks of biochemical components shared by CSKP, CRKP, and PRKP. A *P* value of <0.05 means the difference is statistically significant. All statistical analyses were performed using SPSS statistical software package 22.0 (SPSS, Chicago, IL, USA). Box plots graphically depict groups of numerical data through their quartiles and help display differences between populations or samples.

### Deep learning analysis of SERS spectra.

In order to identify three groups of K. pneumoniae SERS spectral data, this study constructed two deep learning models, CNN and CNN-attention, for the analysis and prediction of bacterial spectra. In order to meet the dimension format of the data required by the neural network algorithm, the expand_dims method in the NumPy (version 1.18.5) library was used to expand the data shape to 3 dimensions. Because it was easier for computers to recognize and process binary data, we used the LabelEncoder function and the to_categorical method in the scikit-learn package (version 0.21.3) to convert the sample labels in the data set into label-encoded forms. The train_test_split function was used to divide all spectral data into training sets, validation sets, and test sets in a 6:2:2 ratio to verify the effectiveness of the model. The test set was completely independent of the training set and validation set and did not participate in the training and validation of the model. In the prepared data set, the shape of each sample input into the neural network was “(1396, 1),” and the shape of the label was “(1, 3).”

For the CNN network architecture, it was mainly composed of input layer, convolution layer, pooling layer, and fully connected layer. When the model started training, the prearranged training and validation data matrices were imported into the input layer. After the input layer, the model included a total of 6 convolutional layers for the extraction of spectral data features. For each convolutional layer in the network, the rectified linear unit (ReLu) nonlinear function was used to improve the nonlinear fitting ability of the neural network. The sizes of the kernels in the convolutional layer were 21*1, 5*1, and 3*1, and the size of the filters was stacked in the form of a pyramid, which increased from small to large values according to the shape of 8, 16, and 64. After every two convolutional layers, we used a maxpooling layer to compress the spectral data dimension in the convolutional operation, thus preventing overfitting and reducing the number of training parameters. The pool_size size of maxpooling was set to 3, 5, and 3, respectively. Before mapping the compressed and dimensionally reduced spectral data into fully connected layers, we used “flatten layers” to stretch and flatten the data into one-dimensional (1D) vectors. Then, the full connection layer (FC) of the network, also known as the classification layer, was used to calculate the probability distribution of feature data. There were 3 neurons in this layer, representing 3 different strains. The Softmax function in the FC layer was used to identify the mapped probability distribution, and the label with the highest probability was selected as the strain result identified by the model. Since Raman fingerprint information was composed of spectral peaks with different wavenumbers and widths, the spectral fingerprints of the same strain were also different in each collection process, which might reduce the accuracy of the combination of different characteristic peaks captured by the CNN model.

In order to enable the CNN model to more accurately aggregate the same type of Raman spectral information, we introduced the attention mechanism and built a CNN-attention model that connected the attention mechanism with CNN in order to enhance the ability of the model to identify important spectral features. Specifically, we embedded our self-written attention function between the last pooling layer and the flatten layer. The attention mechanism used in this study is to directly calculate the spectral feature weights input by the last pooling layer. Inside the mechanism, we built a Softmax function to calculate the probability value. According to the calculated contribution, the attention mechanism will assign more weight to important features. In addition, the optimizer used in the training process of the two network models was Adam, the loss function was categorical_crossentropy, the epoch size was 50, and the batch_size was 128. The model framework was developed based on Python (version 3.7.4), PyCharm (version 2019.3.3, community edition), TensorFlow (version 2.4.1), and Keras (version 2.4.3).

### Evaluation of deep learning analysis.

Different evaluation metrics were used to measure the performance of the two deep learning algorithms, CNN and CNN-attention, via self-written python functions, including accuracy (ACC), loss, precision, recall, and F1 score (f1), which were available under request. In particular, we used the ACC function to calculate the proportion of strain samples that the model correctly predicted among all samples. The loss value was calculated by the categorical_crossentropy loss function. By observing the convergence of the loss value, we could preliminarily assess the quality of the model training. In this study, the changes in ACC and loss values were shown by plotting the learning curves of the two models on the training and validation sets. In order to relatively objectively evaluate the matching degree of the model to the data outside the training set, we used the 5-fold cross-validation method to divide the data into 5 groups (cv = 5) through the cross_val_score function. During each training, a subset of data was selected as the validation set, while the remaining 4 subsets were used as the training set.

To evaluate the training effect of the model, the arithmetic average value of five accuracy points was calculated. In addition, in order to prevent the uneven division of the data set, we used the “Precision” function to calculate the probability that the sample predicted by the model to be a certain strain was actually this type of strain, and the “Recall” function was used to calculate the probability of all strains of a certain type correctly identified by the model. Since “Precision” and “Recall” were a pair of contradictory quantities, we used the f1 function to calculate harmonic mean values. Except for the quantitative evaluation metrics, we also used the receiver operating characteristic (ROC) curve to visualize the performance of the model on the test set. The roc_curve and ROC_auc_score methods in the metrics function were used to calculate the area under the curve (AUC) values. The larger the AUC value or the closer the inflection point of the ROC curve was to the upper left corner, the better the model performance. In order to further show the detailed prediction of the model on the test set data, we used the confusion_matrix function to calculate the probability of the predicted value and the true value of the model, and the output probability values were input into a prewritten plot_confusion_matrix function and visualized as a confusion matrix. The probability that the model correctly predicted each strain was calculated and displayed in the diagonal of the confusion matrix.

### Data interpretability.

In order to intuitively demonstrate the decision-making process of the CNN-attention model on Raman spectra, we developed a significance heatmap for each strain sample by using the self-written Grad-CAM function. When analyzing how the model computed and classified spectral curves, we chose the output of the last convolutional layer as the feature vector because it provided the best balance between high-level semantics and exacted wavenumber information of SERS spectra. Specifically, the model.summary method was used to check the layer name (layer_name) of the last convolutional layer, which, in this study, was “conv1d_5.” In the Grad-CAM function, the models. Model method was used to read the pretrained model and the internal information of the model. The tf.GradientTape method was used to calculate the gradient vector of the last convolutional layer, and the channel mean was calculated using the tf.reduce_mean method. The Grad-CAM of the target strain was generated according to the gradient vector and the channel mean, and the formula was as follows:

$$a_{k}^{c}=\frac{1}{L}\sum_{i=1}^{L}\frac{\partial P_{c}}{\partial F_{\mathrm{kl}}}$$

where *k* represents the kth channel, *l* represents the *l*th Raman shift of the feature vector *F*, and $a_{k}^{c}$ represents the global importance weight of the *k*th feature vector of the Raman spectrum of the target strain.

In addition to obtaining how much the model paid attention to the data features, we also conducted the overall internal analysis process of the data by the model. By calling the TSNE function in Sklearn.manifold to visualize the feature extraction and classification ability of CNN-attention model, the self-written Visual function was used to select the network structure for display. In this study, *the model.summary* method was used to view the number of network layers (num_layer) where each layer structure was located, and the last convolutional layer (num_layer = 8) was selected for visual display. The t-SNE algorithm shows the classification of strain data by the model under different epochs. If strains of the same type were clustered into one group and strains of different types were relatively discrete, it meant that the model had a good classification effect. The TSNE parameters were set to n_components, 2, init, “pca,” and random_state, 0. The plt.text method was used to draw a categorical scatterplot of numerical labels. Strain labels from different categories were grouped together and displayed in different colors.
