# Supplementary material for: Rapid Prediction of Multidrug-Resistant Klebsiella pneumoniae through Deep Learning Analysis of SERS Spectra
Source: Microbiol Spectr. 2023 Mar 6;11(2):e04126-22. doi: 10.1128/spectrum.04126-22 (PMC10100812; doi:10.1128/spectrum.04126-22)
Supplement: Supplemental file 1 — Supplemental material. Download spectrum.04126-22-s0001.pdf, PDF file, 4.2 MB [file spectrum.04126-22-s0001.pdf]

Title

Rapid prediction of multidrug-resistant *Klebsiella pneumoniae* through deep learning analysis of SERS spectra

Running title

Analysis of multidrug-resistant *Klebsiella pneumoniae*

Jing-Wen Lyu [a,b,#], Jia-Wei Tang [c,#], Xue-Di Zhang [a,d,#], Yun-Hu Zhao [b], Su-Ling Liu [b], Yue Zhao [b], Ni Zhang [b], Dan Wang [e], Long Ye [b], Xiao-Li Chen [b], Liang Wang [b,f,*], Bing Gu [a,b*]

[a] Department of Laboratory Medicine, School of Medical Technology, Xuzhou Medical University, Xuzhou, Jiangsu Province, 221000, China

[b] Laboratory Medicine, Guangdong Provincial People's Hospital (Guangdong Academy of Medical Sciences), Southern Medical University, Guangzhou, Guangdong Province, 510080, China

[c] Department of Intelligent Medical Engineering, School of Medical Informatics and Engineering, Xuzhou Medical University, Jiangsu Province, Xuzhou, 221000, China

[d] Laboratory Medicine, The Affiliated Xuzhou Infectious Diseases Hospital of Xuzhou Medical University, Xuzhou, Jiangsu Province, 221000, China

[e] Laboratory Medicine, The Second Affiliated Hospital of Xuzhou Medical University, Xuzhou, Jiangsu Province, 221000, China

[f] School of Medical and Health Sciences, Edith Cowan University, Joondalup, WA, 6027, Australia

[#]These authors have contributed equally to this work and share the first authorship.

[*]Corresponding authors: For all the correspondence, please refer to Prof. Dr. Bing Gu at gubing@gdph.org.cn and Prof. Dr. Liang Wang at wangliang@gdph.org.cn or liang.wang@ecu.edu.au.

Tel: 0086-020-83827812-62462.

Supplementary Table S1 The MICs of Polymyxin B of 121 KP strains. (MIC, μg / ml)

Strains	**Polymyxin B** (BP: I≤2, R≥4)	
	MICs	**Phenotype**
D16	0.25	I
D66-1	0.5	I
D13	0.25	I
D18	0.25	I
D20	0.25	I
D25	0.25	I
D32	0.25	I
D37	0.25	I
D38	0.25	I
D40	0.25	I
D41	0.25	I
D42	0.25	I
D43	0.125	I
D44	0.25	I
D48	0.5	I
D53	0.5	I
D62	0.25	I
D67	0.5	I
D73	0.5	I
D77	0.5	I
D78-1	0.5	I
D78-2	1	I
D79-1	0.5	I
D94	1	I
D95	0.5	I
D96	0.5	I
J1--1	0.25	I
J1--2	0.125	I
J2	0.25	I
J15--2	0.25	I
J22	0.25	I
J26	0.125	I
J28	0.25	I
J39	0.125	I
J46	0.5	I
J47	0.5	I
J48	0.25	I
J49	0.25	I
J50	0.25	I
J54	0.125	I
J59--2	0.5	I

J62	0.5	I
D4	0.25	I
D45	0.25	I
Y30	0.25	I
Y4	0.25	I
Y43	0.25	I
Y39	0.25	I
Y039	0.25	I
Y034	0.25	I
Y3	0.25	I
Y6	0.25	I
Y12	0.25	I
Y14	0.25	I
Y16	0.25	I
Y17	0.25	I
Y18	0.25	I
Y23	0.25	I
Y25	0.25	I
Y26	0.25	I
Y28	0.25	I
Y36	0.125	I
Y37	0.25	I
Y38	0.125	I
Y46	0.125	I
Y47	0.25	I
Y49	0.25	I
Y51	0.25	I
Y53	0.5	I
Y58	0.064	I
Y60	0.25	I
1	0.5	I
2	1	I
3	1	I
7	0.5	I
8	1	I
9	0.5	I
11	0.25	I
12	1	I
13	0.5	I
16	0.5	I
18	0.5	I
19	1	I
23	0.25	I
24	0.5	I
25	0.5	I

29	0.25	I
33	1	I
36	0.25	I
39	0.25	I
43	0.25	I
46	0.5	I
56	0.5	I
58	0.5	I
60	0.25	I
69	0.5	I
71	0.5	I
72	0.5	I
77	0.5	I
79	0.25	I
P1	32	R
P2	32	R
P3	32	R
P4	16	R
P5	32	R
P6	16	R
P7	32	R
P8	64	R
P9	32	R
P10	32	R
P11	8	R
P12	64	R
P13	64	R
P14	32	R
P15	32	R
P16	16	R
P17	32	R
P18	16	R
P19	64	R
P20	64	R
P21	16	R

Abbreviations: MIC, minimum inhibitory concentration; BP, Break point; I, intermediate; R, resistant; S, susceptible

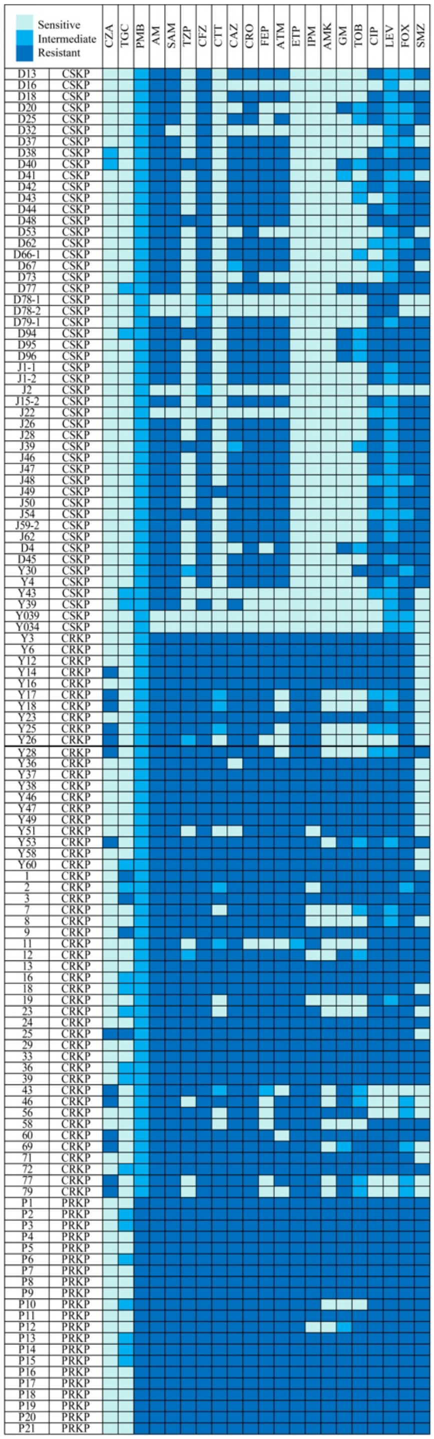

Supplementary Figure S1 Drug susceptibility results of all studied *Klebsiella pneumoniae* strains. The drug susceptibility was divided into three groups: sensitive (light blue), intermediate (blue), and resistance (dark blue).

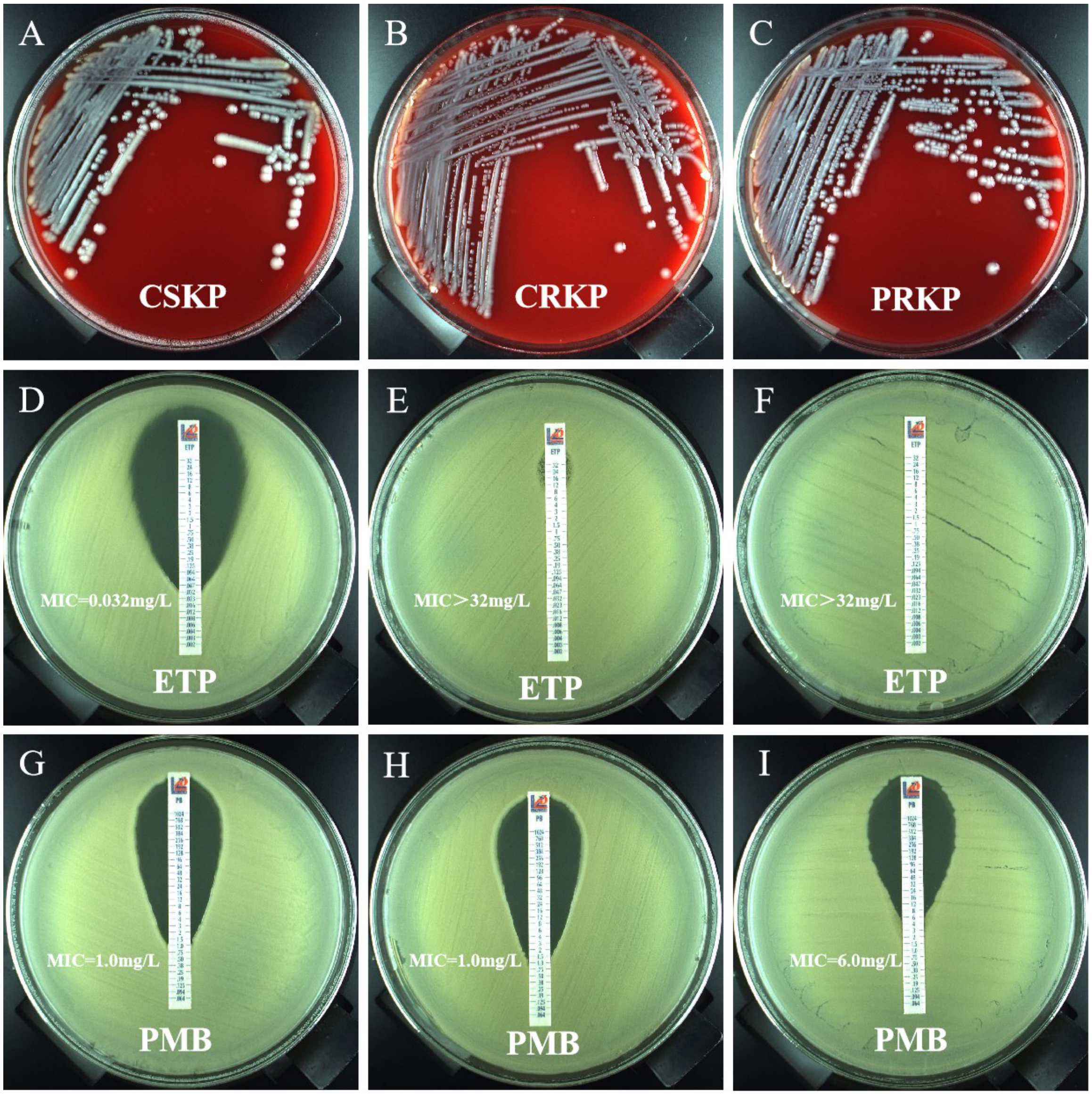

Supplementary Figure S2 Colony morphology and antibiotic resistance results of CSKP, CRKP, and PRKP strains. (A-C) Colony morphologies on Columbia blood agar plates. (D-F) Antibiotic resistance to Ertapenem. (G-I) Antibiotic resistance to Polymyxin B by Liofilchem® MTS.

Supplementary Information

1. Preparation and characterization of silver and gold nanoparticles

1.1 Nanoparticle preparation

AgNPs: 33.72 mg $AgNO_3$ (Sinopharm Chemical Reagent Co., Ltd.) was added to a clean sterile Erlenmeyer flask pre-filled with 200 mL of deionized water (ddH_2O) and heated it on a magnetic stirrer MS-H-ProT (DLAB Pty. Ltd., Beijing, China) to boiling, and then 8 mL of sodium citrate solution (Sinopharm Chemical Reagent Co., Ltd.) was added (1wt%) at one time with stirring at 650 r/min. After heating for 40 minutes, stopped stirring it until the solution temperature up to room temperature (RT). Refill the solution to 200mL with ddH_2O and take 1 mL of the above solution into a 1.5 mL Eppendorf (EP) tube, centrifuge at 7000 r/min for 7 min (Sorvall Legend Micro 21 with 24 x 1.5/2.0mL rotor and lid; 120V 60Hz). discard the supernatant, and resuspend the pellet in 100 μL of ddH_2O to obtain a uniform milky gray solution with negatively charged AgNPs that was stored at 4 ^{o}C away from light until use. AuNPs: add 1 mL of 1wt% $HAuCl_4$ (Sinopharm Chemical Reagent Co., Ltd. China) solution to a clean sterile triangular flask, and then heat it on the magnetic stirrer until boiling. Under the condition of stirring speed of 650 r/min, 1 mL of 1wt% sodium citrate solution was rapidly added. When the color of the solution in the flask changed from light yellow to wine red, continue heating and stirring for 15 minutes, and then stop heating and cool the solution to RT. The final volume was set to 100 mL by adding ddH_2O. 1 mL of the above-prepared solution was then transferred to a 1.5 mL EP and centrifuged at 5600 r/min for 7 min. After the supernatant was removed, store it in the dark at 4 ^{o}C for long-term use.

1.2 Nanoparticle characterization

In order to characterize the sizes and shapes of AgNPs and AuNPs, the absorption spectrum was measured with a UV-vis spectrometer Shimadzu 2600 (Shimadzu Corporation, Japan) in the wavelength range of 300-700 nm. Zeta potential of nanoparticles was measured with Zetasizer Nano ZS 90 (Malvern Instruments Ltd., UK). Transmission electron microscopy (TEM) was conducted by using a FEI Tecnai G2 Spirit BioTWIN TEM with an acceleration voltage of 120 kV. The morphology of the nanoparticles-bacteria mixture was determined by using Scanning Transmission Electron Microscope (STEM) imaging system GeminiSEM (Carl Zeiss NTS Ltd., Japan) that was performed with an acceleration voltage of 2.0 kV.

1.3 Surface-enhanced Raman spectroscopy

Raman spectra for nanoparticles and nanoparticle-bacteria mixture were collected on an inViaTM Raman microscope (Renishaw Plc., New Mills, Wotton-under-Edge, UK) equipped with a 785 nm laser, 1200/mm (514/780) grating, charge-coupled device (Renishaw Centrus 2R4F). Raman shifts ranged from 500 to 1800 cm-1, the exposure time was set to 10 seconds, and the laser power was 0.1%. The Raman system was integrated with a microscope (Leica, Germany), and the laser was coupled through an objective (50×/0.5) to excite the sample and also to collect the scattered Raman signals. The instrument was calibrated with a standard built-in silicon signal at 520 cm-1. 5 µl AgNPs and 5 µl K. pneumoniae solution were mixed on a vortex mixer for 5 seconds, which was then dropped 2.5 µL mixture onto a silicon wafer to dry naturally, and then 64 spots were randomly selected for automatic acquisition of SERS spectra. In particular, map image

acquisition (matrix mode) of Renishau Raman instrument was used to scan points (step=10μm, x=8, y=8). A total of 64 points were auto-scanned, and a large range was randomly selected for matrix scanning for each sample. WiRE 5.3 software (Renishaw plc., Gloucestershire, UK) was used for spectral pretreatment during the analysis.
